# The Discovery of the Human Trypanosome

**Published:** 1902-11-22

**Authors:** Robert Boyce, Ronald Ross


### THE DISCOVERY OF THE HUMAN TRYPANOSOME.

We have received the following communication:—

To the Editors of *The Hospital.*

School of Pathology, Thompson Yates Laboratories,
University College, Liverpool, November 14th, 1902.

Sirs,—In an article on the History of Plague published in the Hospital for November 8th, 1902, it is stated, with reference to the newly found human trypanosome, that it was discovered "within the last few days" at the London School of Tropical Medicine. We trust that you will permit us to give an unqualified denial to this statement. The parasite was discovered in December last by Dr. J. Everett Dutton, of the Liverpool School of Tropical Medicine in a case of Dr. Forde's, Principal Medical Officer of British Gambia, and full accounts of it have already been published by these gentlemen.[1] The London School of Tropical Medicine has no more to do with this discovery than it has to do with the discovery of universal gravitation. We write on behalf of Dr. Dutton, who is now investigating this subject in West Africa. We regret to see the anonymous attempts which are being made in several medical periodicals to misrepresent the facts connected with the history of the discovery.—Yours faithfully,

Robert Boyce, F.R.S., M.B.
Ronald Ross, C.B., F.R.S., F.R.C.S.

[The facts of the case do not justify the tone of the above letter. Whatever may be the history of the original discovery of the trypanosome in question, everyone knows that the fact of its existence in human blood was discovered by Dr. Dutton. We ourselves months ago made a note of its having been

[1] Dutton, Thompson Yates Laboratory Reports, vol. iv., part 2. p. 455, and Brit. Med. Journ., Sept. 20, 1902; Forde, Journ. of Tropical Medicine, Sept. 1, 1902.

found in this connection, and gave the Liverpool School of Tropical Medicine full credit for what had been done. What has now happened is that this parasite, or one allied to it, has been discovered in the blood of a patient at the London School of Tropical Medicine. We did not even announce this as a piece of news, but merely used the fact as an illustration to elucidate our argument on the subject of plague. The statement, however, appears to have been absolutely accurate. Doubtless the London School of Tropical Medicine had nothing to do with the discovery that this trypanosome can exist in human blood, but it had everything to do with the discovery of the parasite in the blood of this patient at the Seamen's Hospital, which was the chief point. There certainly was no misrepresentation of the facts, and, as it seems to us, no excuse for the anger of Professor Boyce and Major Ross.—Ed. The Hospital.]

